# Homeostasis Model Assessment of IR (HOMA-IR) and Metabolic Syndrome (MetS) in First Episode Psychosis

**DOI:** 10.1192/j.eurpsy.2022.1985

**Published:** 2022-09-01

**Authors:** E. Garcia De Jalon, L. Aranguren Conde, A. Aquerreta Unzue, G. Gutiérrez Talavera, A. Corrales Rodriguez

**Affiliations:** 1 SERVICIO NAVARRO DE SALUD, Programa De Primeros Episodios, PAMPLONA, Spain; 2 Complejo Hospitalario de Navarra, Psiquiatría, Pamplona, Spain; 3 SERVICIO NAVARRO DE SALUD, Psiquiatría. Complejo Hospitalario De Navarra, PAMPLONA, Spain

## Abstract

**Introduction:**

Metabolic syndrome (MetS) is common in chronic psychosis but also exists in the early stages. HOMA-IR is an independent predictor of cardiovascular diseases and has already been described in first episode of psychosis.

**Objectives:**

To determine whether HOMA levels differ according to MetS at each time assessment over 2 years.

**Methods:**

MetS and HOMA levels are determined at baseline and at 6, 12, 18 and 24 months in a sample of 50 patients participating in the PEPsNa Early Intervention Programme during two years of follow-up. Adult Treatment Panel III (ATP III) criteria are used to define MetS. Insulin resistance measured with the Homeostatic Model Assessment (HOMA-IR) is computed with the formula fasting plasma glucose (mg/dL) times fasting insulin (mIU/mL) divided by 405. Mann-Whitney U Test are used to compare HOMA variable according to presence of metabolic syndrome.

**Results:**

The results showed that HOMA levels differed statistically significantly between patients who met MetS criteria and those who did not at 12 (p<0.046) and 24 (p<0.004) months of treatment.

**Conclusions:**

Given the small sample size the results of our study indicate that there is a sustained relationship over time between HOMA levels and Metabolic Syndrome (MetS) and that the HOMA IR may be useful in identifying those patients with an increased metabolic and cardiovascular risk.

**Disclosure:**

No significant relationships.

